# Modifying Effect of a Common Polymorphism in the Interleukin-6 Promoter on the Relationship between Long-Term Exposure to Traffic-Related Particulate Matter and Heart Rate Variability

**DOI:** 10.1371/journal.pone.0104978

**Published:** 2014-08-18

**Authors:** Martin Adam, Medea Imboden, Eva Boes, Emmanuel Schaffner, Nino Künzli, Harish Chandra Phuleria, Florian Kronenberg, Jean-Michel Gaspoz, David Carballo, Nicole Probst-Hensch

**Affiliations:** 1 Swiss Tropical and Public Health Institute, Basel, Switzerland; 2 University of Basel, Basel, Switzerland; 3 Division of Genetic Epidemiology, Department of Medical Genetics, Molecular and Clinical Pharmacology, Innsbruck Medical University, Innsbruck, Austria; 4 Department of Community Medicine, Primary Care and Emergency medicine, University Hospitals, Geneva, Switzerland; 5 Cardiology, Department of Internal Medicine Specialties, University Hospitals, Geneva, Switzerland; Utah State University, United States of America

## Abstract

**Background:**

Exposure to particulate matter (PM) has been associated with an increase in many inflammatory markers, including interleukin 6 (*IL6*). Air pollution exposure has also been suggested to induce an imbalance in the autonomic nervous system (ANS), such as a decrease in heart rate variability (HRV). In this study we aimed to investigate the modifying effect of polymorphisms in a major proinflammatory marker gene, interleukin 6 (*IL6*), on the relationship between long-term exposure to traffic-related PM_10_ (TPM_10_) and HRV.

**Methods:**

For this cross-sectional study we analysed 1552 participants of the SAPALDIA cohort aged 50 years and older. Included were persons with valid genotype data, who underwent ambulatory 24-hr electrocardiogram monitoring, and reported on medical history and lifestyle. Main effects of annual average TPM_10_ and *IL6* gene variants (rs1800795; rs2069827; rs2069840; rs10242595) on HRV indices and their interaction with average annual exposure to TPM_10_ were tested, applying a multivariable mixed linear model.

**Results:**

No overall association of TPM_10_ on HRV was found. Carriers of two proinflammatory G-alleles of the functional *IL6* -174 G/C (rs1800795) polymorphism exhibited lower HRV. An inverse association between a 1 µg/m^3^ increment in yearly averaged TPM_10_ and HRV was restricted to GG genotypes at this locus with a standard deviation of normal-to-normal intervals (SDNN) (GG-carriers: −1.8%; 95% confidence interval −3.5 to 0.01; p_interaction(additive)_ = 0.028); and low frequency power (LF) (GG-carriers: −5.7%; 95%CI: −10.4 to −0.8; p_interaction(dominant)_ = 0.049).

**Conclusions:**

Our results are consistent with the hypothesis that traffic-related air pollution decreases heart rate variability through inflammatory mechanisms.

## Introduction

Exposure to air pollution as well as decreased heart rate variability (HRV) as measured by specific indices (SDNN, TP, LF, HF) have been associated with increased cardiovascular morbidity and mortality in longitudinal studies, both in patients with myocardial infarction and in healthy persons [1]–[3]. Heart rate variability (HRV) refers to the beat-to-beat variation in heart rate and is a non-invasive measure of the autonomic regulation of cardiac rhythm [4], [5]. A reduction in different HRV indices can reflect both an increase in sympathetic or a decrease in parasympathetic tone [2]. Several studies have introduced HRV as an intermediate factor between acute exposure to air pollution and cardiovascular morbidity, showing that PM exposure is associated with increased heart rate and reductions in most indices of HRV [6], . A recent meta-analysis including 29 studies supported an inverse relationship between HRV and short-term particulate air pollution [8]. Most studies, including a recent double-blind randomized cross-over study, often focused on acute exposure effects and persons with pre-existing cardiovascular diseases, diabetes mellitus or the elderly [2], [9]. Studies on the chronic impact of air pollution on HRV are scarce [10], [11]. The American Heart Association (AHA) recently stated that studies on long-term effects of air pollution on HRV and cardiovascular health are a major unresolved issue. In a previous study analysing the same study population for the association between long-term TPM_10_ and HRV, we found an impact of air pollution on participants treated for hypertension and heart disease [2].

Air pollution exposure is associated in the short-term with elevated levels of inflammatory markers such as high-sensitivity C-reactive protein (hs-CRP), interleukin 6 (IL-6), fibrinogen and platelet activation, especially in elderly and diabetic subjects, but also in healthy populations [12]–[15]. Long-term circulating interleukin 6 levels in prospectively collected blood are predictive of subsequent risk of coronary heart disease, making *IL6* a strong candidate gene for modifying air pollution effects on coronary heart disease related phenotypes including mortality [16]–[18].

To date, no study has investigated the main association of *IL6* polymorphisms on HRV and their modifying effect on the air pollution-HRV association in the general population. Investigating the modifying effect of genetic variants in interleukin 6 (*IL6*) encoding, a key marker of inflammatory response, on the association between traffic-related air pollution and heart rate variability, can improve the understanding of the mediating role of inflammatory processes. Using data from the “Swiss cohort study on air pollution and lung and heart disease in Adults” (SAPALDIA) we found novel evidence that genetic variation in the pleiotropic *IL6* gene alters heart rate variability and its association with long-term exposure to traffic-related particulate air pollution (TPM_10_).

## Methods

### Ethics statement

The study complies with the declaration of Helsinki and ethical approval for the SAPALDIA study was given by the Swiss Academy of Medical Sciences, the national ethics committee for clinical research (UREK, Project Approval Number 123/00) and the Cantonal Ethics Committees for each of the eight examination areas (Ethics commissions of the cantons Aargau, Basel, Geneva, Grisons, Ticino, Valais, Vaud and Zurich). Participants were required to give written consent before any part of the health examination was conducted either globally (for all health examinations) or separately for each investigation.

### Study population

This study is part of the SAPALDIA cohort which was designed to investigate the long-term health effects of air pollution [19], [20]. In 1991 a random population sample of white adults aged 18–60 years was recruited from eight areas in Switzerland featuring distinct geographic and environmental conditions. While the baseline examination of 9651 persons focused on respiratory health, at follow-up in 2002 (SAPALDIA2) a random sample of in total 1846 persons aged ≥50 years was invited to undergo a 24-hr electrocardiogram (ECG) recording to assess HRV. Exclusion criteria were general or spinal anaesthesia within 8 days (*n* = 5), myocardial infarction within 3 months before the examination (*n* = 2), taking digitalis (*n* = 6), an artificial internal pacemaker (n = 0), and ECG recordings showing atrial fibrillation (*n* = 12), less than 18 hours of recording (*n* = 73) [21] or of insufficient quality (*n* = 6) [22]. From the 1742 participants with 24-hr ECG recordings, we finally included 1552 participants with valid data on cardiovascular risk factors, and TPM_10_ long-term exposure.

### HRV measurements and definition

Measurement of HRV and assessment of cardiovascular risk factors in SAPALDIA have been previously described [22] (Methods S1 in File S1). In brief, participants were asked to follow their regular daily routine during the recording period. Digital devices with a frequency response of 0.05–40 Hz and a resolution of 128 samples/s, recording on three leads (V_1_, altered V_3_ with the electrode on the left mid clavicular line on the lowest rib, and altered V_5_ with the electrode on the left anterior axillary line on the lowest rib) were used. The mean duration of the 24-hr ECG recordings was 22.1 (SD 2.3) hours. The standard deviation of all normal RR (NN) intervals (SDNN) and the following frequency domain variables were calculated: total power (TP) (≤0.40 Hz), ultra-low frequency (ULF) power (≤0.0033 Hz), very low frequency (VLF) power (0.0033–0.04 Hz), low frequency (LF) power (0.04–0.15 Hz), high frequency (HF) power (0.15–0.40 Hz), and the ratio between LF and HF (LF/HF).

### Air pollutant exposure estimation

To assess the effect of long-term exposure to traffic-related pollution on HRV, exposure was defined as the average concentration of traffic-related PM_10_ (TPM_10_) over 10 years. Given that total PM_10_ is not specific to near-road traffic-related pollutants, we focused instead on TPM_10_ to capture the high spatial variability of those source specific pollutants (Methods S1 in File S1). The dispersion modeling approach is described elsewhere [23]. In brief, these Gaussian plume models used the traffic-specific PM_10_ on-road emissions from light- and heavy- duty vehicles, buses, and motorcycles, taking into account diurnal variability, weekday-weekend differences as well as seasonal patterns. Co-variables used in the dispersion models were wind speed and direction, temperature, mixing height, and atmospheric stability classes. By linking participants residential addresses to annual mean TPM_10_ exposure concentrations derived for 200×200 meter grid cells from dispersion models and historical trend data of central site measurements, exposure was individually assigned to all residences of the participants reported for the period between 1990 and 2000 [24]. The PM_10_ exposure modeling and details of the individual exposure assignment have been described before [23], [24]. Since information on short-term TPM_10_ was not available, short-term PM_10_ exposure was used to assess short-term effects on HRV in a sensitivity analysis approach. Short-term PM_10_ was assessed using averaged fixed monitoring station pollution measurements of the same day or up to one week preceding the Holter recording. Data was included from measurement stations nearest to the subjects' home addresses. Subjects living farther than 5 km from a station or having moved within the previous year were excluded for this sensitivity analysis.

### Selection of IL6 genetic variants and genotyping

The single functional nucleotide polymorphism (SNP) in the *IL6* gene locus (rs1800795; *IL6* –174 G/C) was selected as the main candidate to capture the common genetic variation in this chromosomal region. This SNP has shown to be associated with circulating IL-6 blood concentrations [25], [26]. The G allele was identified as the proinflammatory allele in previous epidemiological and experimental studies. In an explorative supplementary analysis benefitting from available GWAS data, three additional *IL6*-SNPs (rs2069827, rs2069840, rs10242595) were included as their genotyping call rates were ≥97.5% and they were haplotype tagging SNPs or associated with cardiovascular phenotypes or in high linkage disequilibrium (LD) with such SNPs (Tables S1–S2 in File S1).

Genomic DNA was isolated from EDTA-buffered whole blood using PUREGENETM DNA Purification Kit (GENTRA Systems, Minneapolis, USA) [19]. Genotypes of three SNPs (rs2069827, rs2069840, rs10242595) were assessed using the Sequenom's MassARRAY system (Sequenom, San Diego, USA) by performing iPLEX single base primer extension and matrix-assisted laser desorption ionization time-of-flight mass spectrometry as described elsewhere [27]. Genotypes of one SNP (rs1800795) were assessed using 5'-nuclease fluorescent realtime PCR (TaqMan) genotyping assay (Applera Europe, Rotkreuz, Switzerland). End-point detection was done using a 7000 ABI System detection device (ABI, Rotkreuz, Switzerland) [28]. For genotyping quality-control, a random selection of >5% of the samples were genotyped twice.

### Statistical analysis

Hardy-Weinberg equilibrium (HWE) was assessed, irrespective of HRV measurement, for the total genotyped SAPALDIA population of 6055 subjects by using the STATA (Table S1 in File S1) genhwi command for global κ-statistic testing. Haploview 4.2 was used for analysis of Lewtonin's linkage disequilibrium (LD) calculating the metrics D-prime and R-squared (Table S2 in File S1).

The SAPALDIA subpopulation for this study is consistent with our previous analyses on the association between TPM_10_ and the log-transformed outcome variable (HRV) [10]. An effect estimate represents percent change in geometric mean. Covariates included in the analysis were chosen in accordance with previous analysis [10] and supplemented by alcohol consumption, passive smoking, diabetes and noise exposure. Covariates included in the regression model were: sex (male as reference), age (years), age^2^, body mass index (bmi, kg/m^2^), bmi^2^, smoking status (never as reference, ever), daily exposure to environmental tobacco smoke (ETS, none as reference, <3 hours, >3 hours), daily alcohol consumption (<1 drink as reference, ≥1 drink), weekly physical activity (to the point of getting out of breath or sweating) (none as reference, between ½ and 2 hours, >2 hours), uric acid concentration (micromol/L), hypertension (no as reference, yes), heart disease (no as reference, yes), diabetes (no as reference, yes), street and railway noise exposure (mean dB(A) per night), seasonal effects (sine and cosine functions of the day of examination with a period of 1 year). High sensitive C-reactive protein (mg/l) was additionally included in the model to guard against potential confounding by unmeasured proinflammatory short-term effects, such as acute air pollution effects. A random intercept in a mixed regression model was included to adjust for potential residual clustering of data within the eight areas of Switzerland.

The underlying genetic model of the SNP effect was defined, using additive and dominance contrasts in the regression models and applying the Cochran-Armitage test to assess potential deviation from the additive model (Methods S1 in File S1). In such a model a significant dominance contrast measure represents deviation from the additive allele effect. To further differentiate in between a dominant and a recessive effect, we considered the direction of the association of the dominance parameter. In the paper we only show p-values of the most likely inheritance mode (additive, dominant or recessive) of the tested main effects of the *IL6* polymorphisms, as well as the interactions of the *IL6* polymorphisms with TPM_10_.

Main effects of *IL6* gene variants were derived from the mixed model described above and adjusted for long-term TPM_10_. *IL6* variants and interaction terms between TPM_10_ and the *IL6* variants were entered into the regression model separately for each genotype. Genotype-specific effect estimates for TPM_10_ were obtained by including the stratum-specific product terms of the genotype with TPM_10_ exposure in covariate-adjusted mixed linear models.

In sensitivity analysis, to control the possible acute effects of ambient air pollution on HRV, we also adjusted the core mixed model for the community average ambient PM_10_-level of the 3 days prior to the Holter recording. Furthermore, we replaced TPM_10_ exposure with the annual NO_2_ exposure averaged over the previous 10 years. In a previous publication we had not found a main effect of NO_2_ on HRV [11].

As this is an exploratory study investigating potential inflammatory mechanisms through a genetic key marker of inflammatory response and a functional SNP in this gene, selected a priori, we did not adjust for multiple testing [29].

All tests were two-sided with a significance level of 0.05. Statistical analyses were performed using STATA, version 12 (StataCorp. 2011. Stata Statistical Software: Release 12. College Station, TX: StataCorp LP) and SAS V 9.2 (SAS Institute, Cary, NC, 2008).

## Results

As described in Table 1, the mean age of the study population was 60.3 (SD 6.2) years with a mean BMI of 26.7 kg/m^2^. Among the subjects, 50.4% were females, 57% ever smokers, 21.5% were exposed to second hand smoke, 46.3% consumed alcohol regularly, 41.2% were physically inactive, 47.7% reported hypertension, 9.5% diabetes and 24.7% a heart disease.

**Table 1:** Characteristics of the study population (N = 1552).

Characteristics	All (n = 1552)	
**Gender**		
Men	770	49.6%
Women	782	50.4%
**Age**	60.3	±6.2
**Lifestyle factors**		
** Smoking status**		
Never Smokers	668	43.0%
Ever Smokers	884	57.0%
**ETS exposure**		
None	1218	78.5%
<3 h/ day	217	14.0%
≥3 h/ day	117	7.5%
**Alcohol**		
<1glass / day	834	53.7%
≥1glass / day	718	46.3%
**Physical acitivity**		
None	640	41.2%
0.5–1.5 h/ week	508	32.7%
≥2 h/ week	404	26.0%
**Cardiovascular health**		
Systolic blood pressure (mmHG)	132.1	±19.1
Diastolic blood pressure (mmHG)	81.7	±10.5
BMI (kg/m2)	26.7	±4.3
**Diabetes**		
No	1404	90.5%
Yes	148	9.5%
**Hypertension**		
No	811	52.3%
Yes	741	47.7%
**Heart disease**		
No	1168	75.3%
Yes	384	24.7%
**Noise Exposure**		
Street noise (dB(A))	38.3	±7.7
Railway noise (dB(A))	6.0	±10.7
**Air pollution**		
Average annual traffic related PM10 (µg/m3)^a^	2.3	±1.3
3-days lag PM10 (µg/m3)^b^	22.0	±14.5
***IL6*** ** polymorphisms**		
** candidate SNP: rs1800795 (−174G/C)**	1549	
GG	584	37.7%
GC	704	45.4%
CC	261	16.8%
**SNPs included in exploratory analyses: rs2069827**	1524	
GG	1264	82.9%
GT	246	16.1%
TT	14	0.9%
** rs2069840**	1526	
CC	638	41.8%
CG	706	46.3%
GG	182	11.9%
** rs10242595**	1529	
GG	664	43.4%
GA	686	44.9%
AA	179	11.7%

ETS indicates environmental tobacco smoke exposure; SBP, systolic blood pressure; DBP, diastolic blood pressure; BMI, body mass index;

a Annual average over 10 years previous to the study.

b average over 3 days previous to the HRV measurement.

The overall analysis including the whole study population (n = 1552) showed no general association of any HRV parameter with TPM_10_ except for the LF/HF ratio which was 3.6% lowered (95% confidence interval (95%CI), −6.9 to −0.2) [10]. The functional *IL6*-174 G/C (rs1800795; n = 1549) polymorphism, which was previously associated with circulating IL-6 [30], [31], was associated with some of the HRV metrics in either an additive or a dominant manner (Table 2). Compared with −174 GG genotypes, participants having only one or no G-risk allele exhibited higher SDNN (GC: 3.8%; 0.8 to 6.8; CC: 4.0%; 95%CI, 0.1 to 8.0; p_additive_ = 0.015) and TP (GC: 7.5%; 95%CI, 0.7 to 14.9; CC: 7.6%; 95%CI, −1.4 to 17.5; p_additive_ = 0.041), while the LF/HF ratio decreased (GC: −8.8%; 95%CI, −14.7 to −2.5; CC: −1.3%; 95%CI, −9.6 to 8.0; p_dominant_ = 0.021). No significant overall association was observed for HF and LF power. None of the other *IL6* SNPs included in the supplementary explorative analysis were statistically significantly associated with HRV (Tables S3–S7 in File S1).

**Table 2:** Unadjusted and adjusted^a^ geometric means of the HRV indices and percent differences in HRV indices by traffic-related PM_10_ (TPM_10_) and by *IL6*-174G/C genotypes (N = 1549).

HRV parameters	*Exposures*	Crude Mean, 95%CI			Adjusted Mean, 95%CI^a^			% change	lower CI	upper CI	p-value^b^	p-value^c^
SDNN	TPM_10_	**131.6**	129.8	133.4	**131.6**	129.9	133.3	**0.08** d	−1.16	1.32	0.905	
	GG	**127.9**	125.1	130.7	**128.6**	125.9	131.4					**0.015 (1)**
	GC	**133.7**	131.0	136.4	**133.3**	130.8	135.9	**3.76** e	0.82	6.79	0.012	
	CC	**134.5**	130.1	139.0	**133.6**	129.4	137.9	**3.98** e	0.07	8.04	0.046	
Total Power (TP)	TPM_10_	**3678.4**	3566.8	3793.4	**3678.4**	3570.7	3789.4	**−0.05** d	−2.76	2.74	0.972	
	GG	**3481.7**	3311.4	3660.8	**3510.9**	3343.8	3686.4					**0.041 (1)**
	GC	**3791.2**	3621.9	3968.4	**3778.6**	3615.0	3949.6	**7.54** e	0.69	14.86	0.030	
	CC	**3825.8**	3549.3	4123.9	**3788.8**	3521.8	4076.1	**7.62** e	−1.42	17.48	0.101	
Low Frequency Power (LF)	TPM_10_	**220.2**	212.0	228.7	**220.2**	212.7	228	−**2.07** d	−5.70	1.71	0.279	
	GG	**212.9**	200.1	226.5	**214.9**	203.0	227.6					**0.296 (1)**
	GC	**220.4**	208.3	233.2	**221.4**	210.2	233.2	**2.68** e	−4.96	10.93	0.503	
	CC	**236.1**	215.2	259.0	**228.3**	209.6	248.8	**5.64** e	−4.71	17.12	0.297	
High Frequency Power (HF)	TPM_10_	**68.6**	65.5	71.8	**68.6**	65.6	71.7	**1.07** d	−3.64	6.00	0.663	
	GG	**63.6**	59.0	68.5	**64.3**	59.7	69.2					**0.116 (1)**
	GC	**72.4**	67.6	77.5	**72.3**	67.6	77.3	**12.70** e	2.01	24.49	0.019	
	CC	**70.2**	62.7	78.4	**68.6**	61.5	76.6	**7.13** e	−6.19	22.35	0.309	
LF/ HF Ratio	TPM_10_	**3.2**	3.1	3.3	**3.2**	3.1	3.3	**−3.60** d	−6.89	−0.19	0.038	
	GG	**3.3**	3.2	3.5	**3.3**	3.2	3.5					**0.021 (2)**
	GC	**3.0**	2.9	3.2	**3.1**	2.9	3.2	**−8.80** e	−14.68	−2.53	0.007	
	CC	**3.4**	3.1	3.6	**3.3**	3.1	3.6	**−1.27** e	−9.66	7.90	0.778	

HRV indicates heart rate variability: SDNN indicates standard deviation of all NN intervals (units ms); TP, total power (ms^2^); HF, high frequency power (ms^2^); LF, low frequency power (ms^2^).

a adjusted for gender, age, age squared, BMI, BMI squared, smoking status, environmental tobacco smoke exposure, alcohol consumption, physical activity, high-sensitivity C-reactive protein, uric acid levels, hypertension, heart disease, diabetes, street and railway noise, seasonal effects and area.

b p-values of genotype-specific main effects of the *IL6*-174G/C polymorphism and TPM_10_ on HRV (codominant genetic model).

c p-values of main effects of the *IL6* -174G/C polymorphism on HRV indices were tested for additive, dominant and recessive genetic models; p-values of the most significant mode of inheritance are presented (^1^additive or ^2^dominant).

d per 1 µg/m^3^ TPM_10_ increase.

e compared to reference genotype G/G.

Table 3 presents the results for the interaction between TPM_10_ exposure and the *IL6*-174 G/C on change in HRV. The *IL6*-174 G/C polymorphism showed interactions with traffic-related PM_10_ and the following HRV metrics: SDNN (p_interaction(additive)_ = 0.028) and LF (p_interaction(dominant)_ = 0.049) (Table 2). A 1 µg/m^3^ increment in yearly averaged TPM_10_ level was associated with a decrease in SDNN of 1.8% (95% confidence interval (95%CI), −3.5 to 0.01) and in LF power of 5.7% (95%CI, −10.4 to −0.8) in participants with the −174 GG genotype. No significant interaction with TPM_10_ exposure and the other HRV metrics (TP, HF power and LF/HF ratio) was observed but for all parameters except HF, TPM_10_ decreased heart rate variability strongest among GG genotype carriers. All other *IL6* SNPs included in the supplementary analysis showed a weaker or no effect modification of TPM_10_ on HRV, respectively, and did not add additional information (Tables S8–S12 in File S1).

**Table 3:** Adjusted^a^ estimates of the mean percent difference of HRV associated with a 1 µg/m^3^ increase in average exposure to traffic-related PM10^b^ by *IL6*-174 G/C genotypes (N = 1549).

HRV parameters	*IL6*-174 G/C	Estimate^a^	95% CI		p_TPM10_ (by genotype)^c^	p_interaction_ (genetic model)^d^
SDNN	GG	**−1.77**	−3.51	0.01	0.051	**0.028 (1)**
	GC	**1.06**	−0.47	2.62	0.177	
	CC	**0.73**	−1.62	3.14	0.545	
Total Power (TP)	GG	**−3.34**	−7.22	0.71	0.105	**0.177 (1)**
	GC	**2.54**	−1.00	6.20	0.161	
	CC	**−1.00**	−6.22	4.51	0.717	
Low Frequency Power (LF)	GG	**−5.70**	−10.36	0.81	0.023	**0.049 (2)**
	GC	**1.51**	−2.87	6.09	0.506	
	CC	**−4.98**	−11.00	1.45	0.126	
High Frequency Power (HF)	GG	**−0.16**	−6.36	6.45	0.961	**0.671 (1)**
	GC	**2.70**	−2.84	8.55	0.346	
	CC	**−3.83**	−11.53	4.54	0.359	
LF/ HF Ratio	GG	**−5.82**	−9.88	1.57	0.008	**0.070 (2)**
	GC	**−1.51**	−5.24	2.37	0.441	
	CC	**−1.54**	−6.98	4.21	0.592	

HRV indicates heart rate variability: SDNN indicates standard deviation of all NN intervals (units ms); TP, total power (ms^2^); HF, high frequency power (ms^2^); LF, low frequency power (ms^2^).

a adjusted for gender, age, age squared, BMI, BMI squared, smoking status, environmental tobacco smoke exposure, alcohol consumption, physical activity, high-sensitivity C-reactive protein, uric acid levels, hypertension, heart disease, diabetes, street and railway noise, seasonal effects and area.

b Annual average over 10 years previous to the study.

c p-values for the genotype- specific TPM_10_ effect estimate.

d p-values of interaction effects of the *IL6*-174 G/C polymorphism with TPM_10_ on HRV were tested for additive, dominant and recessive genetic models; p-values of the most significant mode of inheritance are presented (additive^1^ or dominant^2^).

In order to guard against confounding by short-term exposure of PM_10_, which was not considered in the main and interaction effect model, we included a mean daily PM_10_ exposure measurement over the three days previous to the ECG assessment. Associations reported for long-term exposure to TPM_10_ with *IL6* polymorphisms and their interactions differed only slightly when adjusting additionally for short-term PM_10_ exposure (data not shown). When we replaced TPM_10_ with NO_2_ we observed interactions with *IL6*-174 G/C that were highly comparable to those observed for TPM_10_ (data not shown).

## Discussion

To our knowledge, this is the first report on both, the main effect of genetic variation in *IL6* on HRV and the interaction of *IL6* polymorphisms with air pollution on HRV. In this candidate-approach association study in the general population, we observed the previously described functional *IL6*-174G/C promoter polymorphism to be associated with HRV (SDNN; TP; LF; HF). The association of TPM_10_ with decreased HRV parameters was restricted to carriers of the *IL6*-174 GG genotype. The results lend support to the notion that inflammatory mechanisms mediate part of the air pollution related effects on heart rate variability.

The pleiotropic cytokine IL-6 is central in acute and chronic inflammation by inducing hepatic synthesis of acute phase proteins and by modulating the inflammatory response. In other studies, circulating IL-6 as well as hs-CRP were inversely associated with parasympathetic nervous system tone measured as LF of HRV both, in healthy individuals and patients with CVD or diabetes [32], [33]. Separate lines of evidence provide support to the role of genetic variation in *IL6* in low grade systemic inflammation and, albeit less consistently, cardiovascular disease risk. First, several studies investigated the association of *IL6* gene variants with circulating IL-6 concentrations and with gene activity, most of them focusing on the SNP *IL6*-174 G/C (rs1800795) [30], [31]. The *IL* –174 G-allele, which was associated with lower HRV in this current study was previously associated with higher IL-6 blood concentrations, higher *IL6* gene transcriptional activity, and higher inducible IL-6 responses [34], [35]. The restriction of the TPM_10_ effect on lowering HRV is therefore consistent with an effect in a subgroup predisposed to a proinflammatory state. Second, increased serum levels of repeatedly measured IL-6 were observed among survivors of myocardial infarction who carried the G-allele of this SNP [31]. Third, *IL6* polymorphisms and in particular the −174 G/C variant were previously associated with various cardiovascular disease outcomes and risk factors, including ischemic cerebrovascular events [36], coronary heart disease [37], [38], high blood pressure [37], [39], total cholesterol, LDL, fasting glucose, BMI [40], carotid artery compliance and carotid intima media thickness [39], [41] as well as arterial stiffness and pulse pressure [42]. Yet, in a meta-analysis from 2006 [38] the authors concluded that most of the studies looking at the 174 G/C promoter polymorphism and its association with risk of coronary heart disease (CHD) were case-control studies showing heterogeneous associations between the genotypes and risk of cardiovascular heart disease. In contrast, we excluded all severely ill patients to specifically assess potential effects on HRV in the general population.

Evidence to support the role of systemic low grade inflammation in mediating susceptibility of the autonomic nervous system to air pollution is sparse. A small panel study reported short-term effects of particulate air pollution on decreased HRV to be stronger among elderly persons with high levels of CRP and fibrinogen [43]. While studies on the interaction between air pollution and inflammatory gene variants may be better suited for assessing susceptibility to long-term exposures, data on gene-air pollution interactions in cardiovascular health area generally very sparse as recently reviewed by Zanobetti et al. [44]. In fact, all results published on gene-air pollution interactions in relation to HRV were derived from a single study, the Normative Ageing Study of men, and focused on acute effects of air pollution. Results from this study generally support the role of pulmonary or systemic oxidative stress in linking air pollution and HRV [45], [46]. The SAPADIA cohort team previously reported on the modifying effect of antioxidative *GST* gene polymorphisms (*GSTM1* and *GSTT1* gene deletions and the *GSTP1* SNP Ile105Val) in the association of HRV with the inflammatory risk factors second-hand smoke and obesity in the general population [47]. These *GST* polymorphisms did not modify the association between TPM_10_ and HRV in this study.

A major strength of our study is the population-based design and the detailed information available on participants. The exposure assessment of TPM_10_ with individual exposure estimates taking residential history into consideration has advantages to assess long-term exposure to traffic-related air pollution, providing good differentiation. Due to the detailed information on numerous cardiovascular risk factors, we were able to control for major confounding factors. Furthermore, we were able to guard our analysis of long-term TM_10_ effects against confounding by short-term PM_10_ exposure. The likely absence of confounding by short-term air pollution is further supported by the fact that non-inclusion of high sensitive C-reactive protein as model covariate did not materially alter the results (Tables S13-S14 in File S1).

This study has also a number of limitations. First, the results presented refer to statistical associations and interactions. The low prevalence of some genotypes limited statistical power of the explanatory analyses. Calculations indicate we had sufficient power to observe a main effect and a TPM_10_ interaction effect for the candidate SNP *IL6*-174 G/C on SDNN of the order of magnitude reported or larger. These power calculations assumed different HRV parameters to not be independent. Second, we did not have information about IL-6 serum levels and cannot draw conclusions on how the polymorphisms studied influenced the circulating IL-6 levels and finally the cardiovascular health of the SAPALDIA participants. But the *IL6*-174 G/C SNP was associated with higher high sensitive C-reactive protein (mg/l) concentrations in the blood (GG: 2.8; GC: 2.5; CC: 2.4). Third, data on short-term TPM_10_ was not available, and hence short-term exposure was assessed using averaged normal PM_10_ from fixed monitoring station measurements. We had obtained 24-hr ECG recordings only once for each participant and were therefore not able to look at longitudinal changes in HRV and its association with the incidence of cardiovascular diseases. Fourth, we had previously reported that the association between TPM_10_ and HRV is restricted to persons reporting intake of angiotensin converting enzyme inhibitors (ACEI) [10]. The now reported interaction between the *IL6*-174G/C polymorphism and TPM_10_ remained unchanged after exclusion of participants on ACEI, but we lacked the statistical power for formal assessment of 3-way interactions between TPM_10_, *IL6* variants and ACEI. Finally, we were unable to adjust our analysis for some short-term factors influencing HRV, such as physical exercise. However, physical exercise at the time of heart rate variability measurement is unlikely to be associated with chronic exposure to TPM_10_ and thus unlikely to confound the associations reported.

## Conclusions

In summary, this cross-sectional study from the general population provides supportive evidence that genetic variation in one of the major proinflammatoy cytokines, *IL6*, alters HRV and its association with long-term exposure to traffic-related particulate air pollution. Our findings contribute to the research effort to pinpoint biologic mechanisms mediating air pollution related broad health effects. While the results guide future research efforts to use *IL6* as an important candidate gene in studies of HRV and air pollution, they essentially need replication in different study populations, in longitudinal studies, for different air pollution exposure metrics, and with genetic variant information derived from deep sequencing of the *IL6* gene region.
